# Genome-wide study on genetic diversity and phylogeny of five species in the genus *Cervus*

**DOI:** 10.1186/s12864-019-5785-z

**Published:** 2019-05-17

**Authors:** Pengfei Hu, Yuanchen Shao, Jiaping Xu, Tianjiao Wang, Yiqing Li, Huamiao Liu, Min Rong, Weilin Su, Binxi Chen, Songhuan Cui, Xuezhe Cui, Fuhe Yang, Hidetoshi Tamate, Xiumei Xing

**Affiliations:** 1 State key laboratory for molecular biology of special economic animals, Key laboratory of genetics, breeding and reproduction of special economic animals, Institute of special animal and plant sciences, Chinese academy of agricultural sciences, Changchun, China; 2 Animal Health Supervision Institute of Hainan Province, Haikou, China; 3 Faculty of Science, Yamagata University, Yamagata, Japan

**Keywords:** Phylogeny of *Cervus*, Reduced-representation genome sequencing, Interspecific gene exchange, Genetic diversity

## Abstract

**Background:**

Previous investigations of phylogeny in *Cervus* recovered many clades without whole genomic support.

**Methods:**

In this study, the genetic diversity and phylogeny of 5 species (21 subspecies/populations from *C. unicolor*, *C. albirostris*, *C. nippon*, *C. elaphus* and *C. eldii*) in the genus *Cervus* were analyzed using reduced-representation genome sequencing.

**Results:**

A total of 197,543 SNPs were identified with an average sequencing depth of 16 x. A total of 21 SNP matrices for each subspecies/population and 1 matrix for individual analysis were constructed, respectively. Nucleotide diversity and heterozygosity analysis showed that all 21 subspecies/populations had different degrees of genetic diversity. *C. eldii*, *C. unicolor* and *C. albirostris* showed relatively high expected and observed heterozygosity, while observed heterozygosity in *C. nippon* was the lowest, indicating there was a certain degree of inbreeding rate in these subspecies/populations. Phylogenetic ML tree of all *Cervus* based on the 21 SNP matrices showed 5 robustly supported clades that clearly separate *C. eldii*, *C. unicolor*, *C. albirostris*, *C. elaphus* and *C. nippon*. Within *C. elaphus* clade, 4 subclades were well differentiated and statistically highly supported: *C. elaphus* (New Zealand), *C. e. yarkandensis*, *C. c. canadensis* and the other grouping the rest of *C. canadensis* from China. In the *C. nippon* clade, 2 well-distinct subclades corresponding to *C. n. aplodontus* and other *C. nippon* populations were separated. Phylogenetic reconstruction indicated that the first evolutionary event of the genus *Cervus* occurred approximately 7.4 millions of years ago. The split between *C. elaphus* and *C. nippon* could be estimated at around 3.6 millions of years ago. Phylogenetic ML tree of all samples based on individual SNP matrices, together with geographic distribution, have shown that there were 3 major subclades of *C. elaphus* and *C. canadensis* in China, namely *C. e. yarkandensis* (distributed in Tarim Basin), *C. c. macneilli/C. c. kansuensis*/*C. c. alashanicus* (distributed in middle west of China), and *C. c. songaricus*/*C. c. sibiricus* (distributed in northwest of China). Among them, *C. e. yarkandensis* was molecularly the most primitive subclade, with a differentiation dating back to 0.8–2.2 Myr ago. D statistical analysis showed that there was high probability of interspecific gene exchange between *C. albirostris* and *C. eldii*, *C. albirostris* and *C. unicolor*, *C. nippon* and *C. unicolor*, and there might be 2 migration events among 5 species in the genus *Cervus*.

**Conclusions:**

Our results provided new insight to the genetic diversity and phylogeny of *Cervus* deer. In view of the current status of these populations, their conservation category will need to be reassessed.

**Electronic supplementary material:**

The online version of this article (10.1186/s12864-019-5785-z) contains supplementary material, which is available to authorized users.

## Background

The *Cervidae* phylogeny has proven difficult to resolve, due either to rapid radiation in Pecorans (superordinate clade of *Cervide*) in the Mid-Eocene or ambiguity and conflict between different analyses [1–3], especially phylogenetic relationships among formerly included in *Cervus* (*Rusa, Rucervus, Przewalskium*, *Panolia* and *Cervus*). The recent publication [4], showed that several species should be classified into the genus *Cervus*: *Elaphurus davidianus*, *Przewalskium albirostris*, *Rucervus eldii*, and all species of *Rusa*, and sometimes *Panolia eldii* was wrongly included in the genus *Rucervus*, the genetic analysis demonstrated the significant evolutionary and systematic distance between *Panolia eldii* and *Rucervus duvaucelii*. They were approved by some important publications [5, 6]. While some zoologists hold different opinions based on previous studies in the year 2001–2008 [7, 8]. At present, it is generally considered that there are 6 or 7 closely related taxa in the genus *Cervus*, namely *C. timorensis*, *C. unicolor*, *C. albirostris*, *C. nippon*, *C. elaphus*, *C. canadensis* and *C. eldii*. Past taxonomic studies, based on morphology [9], karyotypes [10, 11], serum proteins [12], mitochondrial DNA markers [13–22] and/or microsatellite DNA markers [23, 24] have led to partial consensus, reflecting the sparse amount of phylogenetic information available for some of these taxa, and disagreements between comparative anatomy and molecular systematics [1, 3].

Karyotype analysis showed that *C. unicolor* was the most primitive species in the genus *Cervus*, followed by *C. albirostris* and *C. nippon*, and then *C. elaphus, C. eldii* has close relationship with *C. unicolor* [11]. Based on the combined analysis of mitochondrial and nuclear genes, the genus *Cervus* was found polyphyletic, *C. eldii* form sister group to the clade plus all other species of *Cervus* (*C. unicolor*, *C. albirostris*, *C. elaphus* and *C. nippon*) [5]. Some researchers believed that *C. elaphus* and *C. canadensis* were derived from a single common ancestor, while *C. elaphus* form a sister group to *C. canadensis* and *C. nippon*, *C. canadensis* share more nucleotide similarities with *C. nippon* [19], while other researchers presumed that *C. nippon* is more primitive than *C. elaphus* and *C. canadensis* [13, 16, 17], eight subspecies of the *C. elaphus* probably originated from *C. nippon* in the middle Pleistocene [14, 25–27], however, *C. nippon* is allied with *C. elaphus* in nuclear gene analysis [5]. The Ag-No number and sites of *C. nippon*, *C. elaphus* and *C. canadensis* were same in karyotype analysis [11], the C-band and G-band were similar. Actually, the fawn of *C. nippon*, *C. elaphus* and *C. canadensis* were all spotted white, and there is no reproductive isolation between them. The above results imply that *C. elaphus*, *C. canadensis* and *C. nippon* could be very close-related species in the genus *Cervus*. Nonetheless, the classification proposed by all these studies are not in concordance with each other suggesting more work is needed to clarify these relationships.

The specific aims of our study were (1) to investigate the phylogeny of the genus *Cervus*, (2) to test the genetic diversity in the *Cervus* populations, and (3) to investigate interspecific gene exchange between *C. unicolor*, *C. albirostris*, *C. nippon*, *C. elaphus* and *C. eldii*.

To achieve these aims, SNP data from reduced-representation genome sequencing were used to provide phylogenetic relationships among 21 subspecies/populations derived from 5 species of genus *Cervus*, the divergence times, interspecific gene exchange and genetic diversity were estimated.

## Methods

### Ethics statement

All procedures concerning animals were organized to accord with the guidelines of care and use of experimental animals established by the Ministry of Agriculture of China, and all protocols were approved by the Institutional Animal Care and Use Committee of Institute of Special Animal and Plant Sciences, Chinese Academy of Agricultural Sciences, Changchun, China.

### Samples

**Fig. 1:**
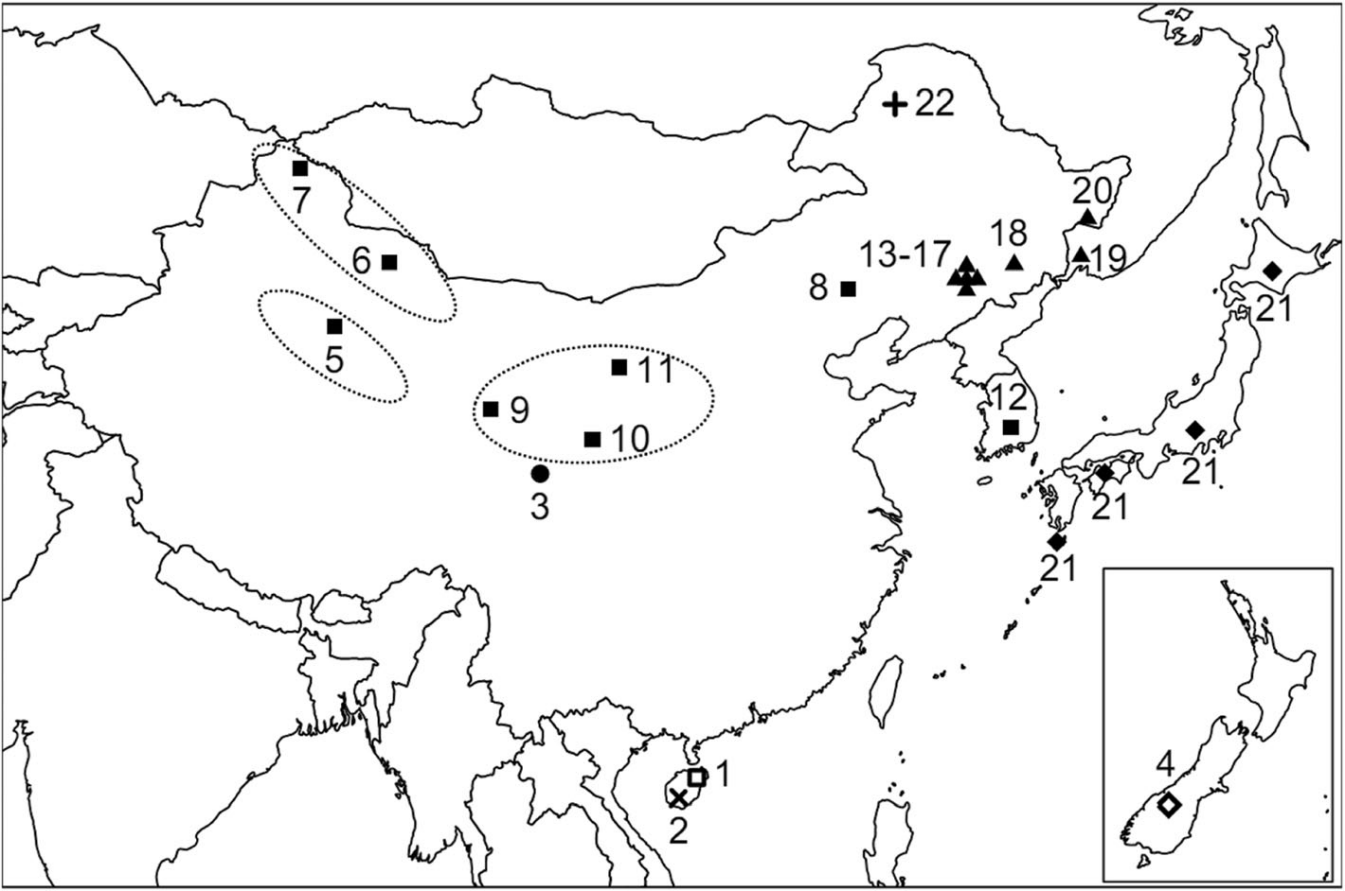
Map showing approximate sample collection sites. Numbers next to sites are equivalent to numbers in Table 1. Symbols for subspecies are identical to group symbols in Table 1. The lower right corner of the map is the geographic distribution of samples from New Zealand. Dotted elliptical circles represent three main subclades of *C. elaphus* and *C. canadensis* in China. The map was taken from maps package in R 3.2.0 software, we acknowledge its offer

We sequenced a total of 195 samples, including 185 samples from 21 subspecies/populations, representing the 5 described species of *Cervus* (*C. eldii, C. unicolor, C. albirostris, C. elaphus, C. nippon*), and 10 samples from *Rangifer* (*R. tarandus*) as out-group. The information about species distribution, classification, source, and number was collected (Fig. 1 and Table 1). All samples, apart from *C. n. aplodontus* that were wild-caught, were raised in captivity, all of them are derived from wild-caught deer and have been maintained under closed flock breeding for a range of 5–50 generations. Chemical anesthesia was used during deer catching, Lumianning injection (070011777, Jilin Huamu Animal Health Products Co., Ltd., China), an anesthetic, was administered intramuscularly 1 ml per 100 kg body weight, peripheral vein blood of each sample were collected fresh and stored in − 20 °C until DNA extraction.

**Table 1:** Samples analyzed in this study

Nr.	Subspecies/populations	*N*	Geographical provenance
1	*C. eldii* (*Panolia eldii*) McClelland, 1842	7	Hainan, China
2	*C. unicolor* Kerr, 1792	5	Hainan, China
3	*C. albirostris* Przewalski, 1883	9	Qinghai, China
4	*C. elaphus* (New Zealand)	11	New Zealand
5	*C. e. yarkandensis* Blanford, 1892	9	Tarim Basin, China
6	*C. c. songaricus* Severtzov, 1873	10	Tien Shan, China
7	*C. c. sibiricus* Severtzov, 1873	9	Altai, China
8	*C. c. xanthopygus* Milne-Edwards, 1867	11	Inner Mongolia, China
9	*C. c. macneilli* Lydekker, 1909	9	Qinghai, China
10	*C. c. kansuensis* Pocock, 1912	10	Kansu, China
11	*C. c. alashanicus* Bobrinskii and Flerov, 1935	3	Helanshan, China
12	*C. c. canadensis* Erxleben, 1777	7	South Korea
13	*C. n. hortulorum* Swinhoe, 1864 (shuangyang)	9	Shuangyang, China
14	*C. n. hortulorum* Swinhoe, 1864 (tonghua)	9	Tonghua, China
15	*C. n. hortulorum* Swinhoe, 1864 (dongda)	10	Dongda, China
16	*C. n. hortulorum* Swinhoe, 1864 (dongfeng)	10	Dongfeng, China
17	*C. n. hortulorum* Swinhoe, 1864 (siping)	10	Siping, China
18	*C. n. hortulorum* Swinhoe, 1864 (aodong)	11	Aodong, China
19	*C. n. hortulorum* Swinhoe, 1864 (ussuri)	6	Vladivostok, Russia
20	*C. n. hortulorum* Swinhoe, 1864 (xingkaihu)	9	Xingkaihu, China
21	*C. n. aplodontus* Heude, 1884	11	Japan (Yakushima, Kyushu, Honshu, Hokkaido)
22	*R. tarandus* Hamilton Smith, 1827	10	Inner Mongolia, China

*C.*, *Cervus*; *C. e.*, *C. elaphus*; *C. c.*, *C. canadensis*; *C. n.*, *C. nippon*; *R., Rangifer*; *N*, number of individual samples of population

### Genomic DNA extraction

Genomic DNA extraction was carried out using whole blood genome DNA isolation kit (BioTeke Corporation, Beijing, China). The degradation and contamination of the genomic DNA was monitored on 1% agarose gels, DNA purity was checked using the NanoPhotometer® spectrophotometer (IMPLEN, CA, USA). DNA concentration was measured using Qubit® DNA Assay Kit in Qubit® 2.0 Flurometer (Life Technologies, CA, USA).

### Library preparation and Illumina sequencing

We carried out a pre-design experiment, the restriction sites of Mse I, Nla III and Hae III in the reference genome assembly (mhl.quiver-7-1) were predicted, the result showed that the three enzyme approach is more favorable than one enzyme or two enzyme method in library construction for the reduced-representation genome sequencing. The average enzyme capture rate was 97.0% (Additional file 1: Table S1). So the three enzyme approach was selected in formal experiments. Genomic DNA was incubated at 37 °C with Mse I (New England Biolabs, NEB), T4 DNA ligase (NEB), ATP (NEB), and Mse I Y adapter N containing barcode. Restriction ligation reactions were heat-inactivated at 65 °C, and then digested for additional restriction enzyme Nla III (NEB) and Hae III (NEB) at 37 °C. The restriction ligation samples were purified with Agencourt AMPure XP (Beckman), then performed PCR reaction using purified samples, Phusion Master Mix (NEB) universal primer and index primer to add index, complete i5 and i7 sequence. The PCR productions were purified using Agencourt AMPure XP (Beckman) and pooled, then run out on a 2% agarose gel. Fragments with 400–425 bp (with indexes and adaptors) in size were isolated using a Gel Extraction Kit (Qiagen). These fragment products were then purified using Agencourt AMPure XP (Beckman), which was diluted for sequencing. Pair-end sequencing was performed upon the selected tags using an Illumina PE150 high throughput sequencing platform at the DNA sequencing laboratory (Tianjin Novogene Bioinformatics Technology Co., Ltd).

### Read mapping and quality control

The sequences of each sample were sorted according to the barcodes. To make sure reads reliable and without artificial bias in the following analyses, raw data of fastq format was firstly processed through a series of quality control (QC) procedures in-house C scripts. QC standards as the following: (1) Removing reads with ≥10% unidentified nucleotides (N); (2) Removing reads with > 50% bases having phred quality < 5; (3) Removing reads with > 10 nt aligned to the adapter, allowing ≤10% mismatches. The assembly sika deer genome (mhl.quiver-7-1) was taken as reference genome (unpublished data). Comparison analysis of each sample was performed using BWA software (Parameter: mem -t 4 -k 32 -M -R).

### SNP matrix construction

Population SNPs detection was performed with Stacks based on the clean data, the SNP datasets were filtered with the following criterion: maf = 0.01, mis = 0.7, dp = 2. For the population-based analysis, according to the clustering information (Table 1), the SNP information of each population is extracted from the filtered VCF file, and discarded those SNPs that may represent duplicated regions in the genome, finally the SNP matrix of each population is obtained. For the individual-based analysis, we constructed a SNP matrix with 197,543 SNP sites based on Stacks result.

### Genetic diversity analysis

Nucleotide diversity (π) reflects population polymorphism, it was calculated using Arlequin software (http://cmpg.unibe.ch/software/arlequin35/). Expected heterozygosity (He) and Observed heterozygosity (Ho) were calculated according to the formula provided by Nei [28].

### Phylogenetic analysis

Phylogenetic analyses were conducted using maximum likelihood (ML) on inter-population SNP matrix and individual SNP matrix, respectively. All ML analyses were conducted using the on line version of RAxML program [29] with the general time-reversible and gamma model (GTR + G model) of sequence evolution. The topology was evaluated with 1000 bootstrap replicates. A Yule speciation process was implemented as the tree prior and 1 X 10^6^ steps were used in the Markov chain Monte Carlo (MCMC) iterations with sampling every 1000 iterations. Divergence times were estimated using MCMCTree 4.9e program (http://abacus.gene.ucl.ac.uk/software/paml.html), divergence time for *R. tarandus* and *C. n. aplodontus* 13.60–13.88 millions of years (http://www.timetree.org/home) were used as time correction point, the results were visualized using FigTree software.

### Gene exchange analysis

Interspecific gene exchange of *C. eldii, C. unicolor, C. albirostris, C. elaphus and C. nippon* were analyzed by D-statistics using AdmixTools software. D-statistics is a method included in AdmixTools to analyze the admixture of ancestral population. It infers the distances of genetic relationships between populations by determining gene exchange or gene infiltration between populations. This method usually takes four populations W, X, Y and Z as analysis objects, among them, Z is an exogenous group and Y is an ancestral group. If the analysis showed that there were gene flows between W and Y, X and Z, the BABA model is fitted, so the relationship between W and Y is closer; If there is gene flow between W and Z, X and Y, the ABBA model is fitted, so the relationship between X and Y is closer. In this study, for SNP locus i, the allele frequencies of different populations were w’, x’, y’, z’, respectively. Firstly, BABA was defined, which meant that the alleles of W and Y were the same, the alleles of X and Z were the same, and the alleles of W and X were different. Similarly, ABBA was defined, then gene exchange analysis was carried out accordingly. Population differentiation and mixing were estimated by TreeMix based on genome-wide allele frequency, 2–5 gradients were set respectively, combined with D statistical results, calculated the number of migration events among 5 species [30].

## Results

### Reduced-representation genome sequencing

The sequencing of 195 individuals resulted in a total of 84,231,376,992 bp raw de-multiplexed reads with an average 431,955,779 bp reads per individual. After strict filtration, high quality clean data were obtained. All the sequencing data have been statistical analyzed, including output, error rate, Q20, Q30, GC content (Additional file 2: Table S2). The raw sequencing reads were deposited in NCBI SRA (Accession numbers: PRJNA355630). We successfully mapped 97.51% of reads per sample to the reference genome. The average estimated site coverage per sample was 16.21 x. The average 1 x genome coverage rate was 3.00% (Additional file 3: Table S3).

### SNP matrices

**Fig. 2:**
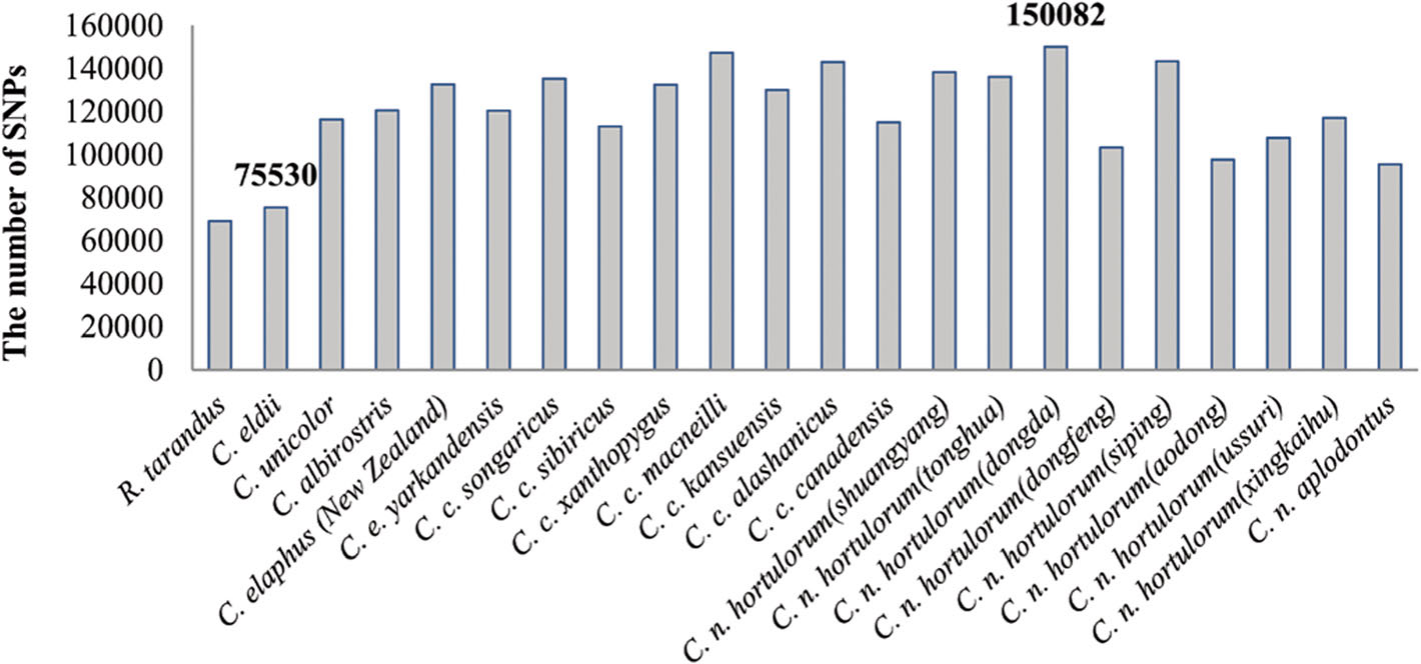
Number of SNPs among 21 subspecies/populations. Population SNPs detection was obtained by Stacks based on the clean data. The average number of SNPs among 21 subspecies/populations was 120,030, *C. nippon* (dongda) had the largest number of SNPs (150,082 SNPs), *C. eldii* had the least number of SNPs (75,530 SNPs) in *Cervus*, followed by *C. n. aplodontus* (95,583 SNPs)

Under inter-population combined analysis, we generated a total of 21 SNP matrices for the phylogenetic analyses. The average number of SNPs among 21 subspecies/populations was 120,030 (Fig. 2), *C. nippon* (dongda) had the largest number of SNPs (150,082 SNPs), *C. eldii* had the least number of SNPs (75,530 SNPs) in *Cervus*, followed by *C. n. aplodontus* (95,583 SNPs). In all populations, the number of SNPs in *R. tarandus* was the least (69,103 SNPs). Individual analysis produced a matrix containing 197,543 SNPs.

### Genetic diversity analysis

Nucleotide diversity (π) ranged from 0.009 to 0.022 among 21 subspecies/populations, with *C. nippon* (aodong) having the lowest (0.009) and *C. c. alashanicus* having the highest (0.022) π average (Table 2). Furthermore, we found that He was higher than other subspecies/populations in *C. c. alashanicus* and *C. eldii* (0.573 and 0.542, respectively). Subspecies/populations in *C. elaphus* (New Zealand) and *C. c. xanthopygus* showed relatively low average He (0.373 and 0.388, respectively) compared to other species of *Cervus*, and Ho was higher than other subspecies/populations in *C. eldii* and *C. albirostris* (0.632 and 0.396, respectively), while subspecies/populations in *C. nippon* (aodong) and *C. nippon* (xingkaihu) showed relatively low average Ho (0.103 and 0.106, respectively). Generally, all 21 subspecies/populations showed different degrees of genetic diversity. *C. eldii*, *C. unicolor* and *C. albirostris* showed relatively high He and Ho, while Ho in *C. nippon* was the lowest, indicating there was a certain degree of inbreeding rate in these subspecies/populations.

**Table 2:** Population genetic diversity analysis of 22 subspecies/populations

	Nucleotide diversity (π)	Expected heterozygosity (He)	Observed heterozygosity (Ho)
*C. eldii*	0.01195	0.54208	0.63219
*C. unicolor*	0.01723	0.48818	0.28407
*C. albirostris*	0.01264	0.43885	0.39629
*C. elaphus* (New Zealand)	0.01117	0.37294	0.11788
*C. e. yarkandensis*	0.01214	0.41992	0.16645
*C. c. songaricus*	0.01127	0.40494	0.12928
*C. c. sibiricus*	0.01100	0.42018	0.12988
*C. c. xanthopygus*	0.01019	0.38841	0.12362
*C. c. macneilli*	0.01193	0.40026	0.12525
*C. c. kansuensis*	0.01171	0.40866	0.13240
*C. c. alashanicus*	0.02236	0.57279	0.23398
*C. c. canadensis*	0.01258	0.44756	0.16953
*C. n. hortulorum* (shuangyang)	0.01080	0.42691	0.10598
*C. n. hortulorum* (tonghua)	0.01050	0.42977	0.11040
*C. n. hortulorum* (dongda)	0.01211	0.40347	0.10471
*C. n. hortulorum* (dongfeng)	0.01035	0.41366	0.10944
*C. n. hortulorum* (siping)	0.01201	0.40177	0.10735
*C. n. hortulorum* (aodong)	0.00921	0.42030	0.10332
*C. n. hortulorum* (ussuri)	0.01303	0.48392	0.13704
*C. n. hortulorum* (xingkaihu)	0.01144	0.42425	0.10557
*C. n. aplodontus*	0.00995	0.40415	0.12920
*R. tarandus*	0.01177	0.44991	0.50521

### Phylogeny of *Cervus*

**Fig. 3:**
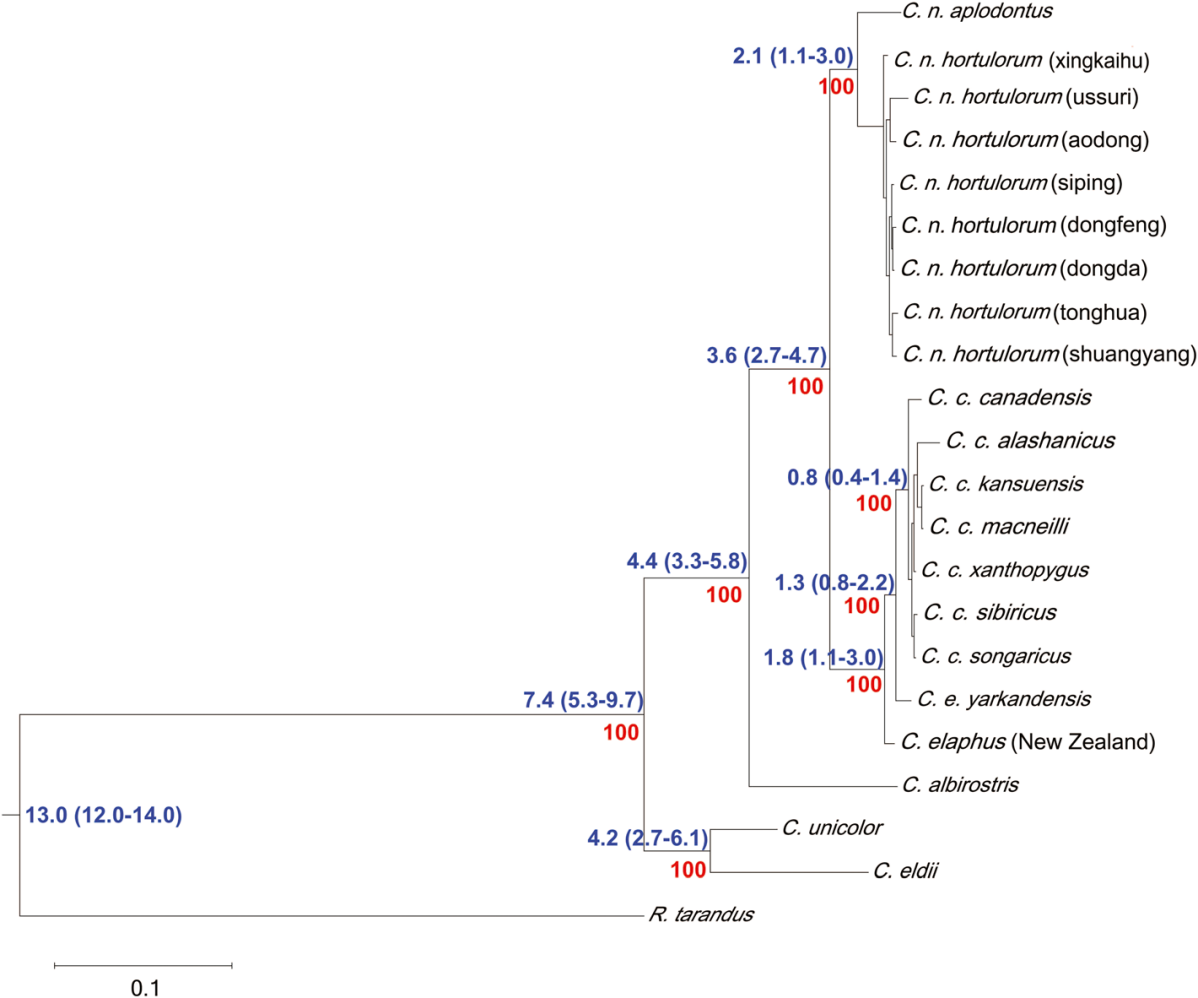
Phylogenetic ML tree and divergence time analysis of all *Cervus* clades based on the 21 SNP matrices. Phylogenetic analyses were conducted using maximum likelihood (ML) on inter-population SNP matrix, divergence times were estimated using MCMCTree 4.9e program, divergence time for *R. tarandus* and *C. n. aplodontus* 13.60–13.88 millions of years was used as time correction point. Phylogenetic ML tree of all *Cervus* clades based on the 21 SNP matrices showed that 5 clades that clearly separate *C. eldii, C. unicolor, C. albirostris, C. elaphus* and *C. nippon* with the highest bootstrap support. Our phylogenetic reconstruction indicated that the first evolutionary event of the genus *Cervus* occurred approximately 7.4 millions of years ago

Phylogenetic ML tree of all *Cervus* clades based on the 21 SNP matrices showed that 5 clades that clearly separate *C. eldii*, *C. unicolor*, *C. albirostris*, *C. elaphus* and *C. nippon* from 21 subspecies/populations with the highest bootstrap support (Fig. 3). Within *C. elaphus* clade, 4 subclades were well differentiated and statistically highly supported: *C. elaphus* (New Zealand), *C. e. yarkandensis*, *C. c. canadensis* and the other grouping the rest of *C. canadensis* from China. In the *C. nippon* clade, 2 well-distinct subclades corresponding to *C. n. aplodontus* and other *C. nippon* populations were separated. Our phylogenetic reconstruction indicated that the first evolutionary event of the genus *Cervus* occurred approximately 7.4 millions of years ago. The divergence from a common ancestor of *C. unicolor* and *C. eldii* would have occurred at 2.7–6.1 millions of years (Myr) ago, so *C. unicolor* and *C. eldii* might be the most primitive *Cervus* deer. *C. albirostris* was the closest to them with divergence time of 3.3–5.8 Myr ago. The split between *C. elaphus* and *C. nippon* could be estimated at around 3.6 millions of years ago, the results were consist with previous report [6]. *C. elaphus*, *C. canadensis* and *C. nippon* might have the same ancestor. It is surprising because *C. elaphus* (New Zealand) and *C. e. yarkandensis* were sisters to *C. canadensis* while usually *C. canadensis* turn out to be more closely related to *C. nippon* than to *C. elaphus*.

In the *C. elaphus* clade, the divergence between *C. elaphus* (New Zealand) and the other would have occurred at 1.1–3.0 Myr ago, it was accordant with fossil record [31]. The separation of *C. e. yarkandensis* and *C. c. canadensis* took place more recently, nearly 0.8–2.2 and 0.4–1.4 Myr ago. Whereas the bootstrap support of divergence was not high among other *C. canadensis* populations. In the *C. nippon* clade, there was a distinct divergence between *C. n. aplodontus* and other *C. nippon* populations, and the divergence time between them was 1.1–3.0 Myr. The divergence of other *C. nippon* populations was not obvious, so *C. n. aplodontus* might be the most primitive subspecies of *C. nippon* in our study.

### Three main subclades of *C. elaphus* and *C. canadensis* in China

The *C. elaphus* and *C. canadensis* were geographically widespread in history. At present, it was considered that there were 7 or 8 subspecies of *C. elaphus* and *C. canadensis* lived in mainland China, and they were very restricted in distribution. All the subspecies except *C. c. wallichii* were collected and analyzed in this study.

**Fig. 4:**
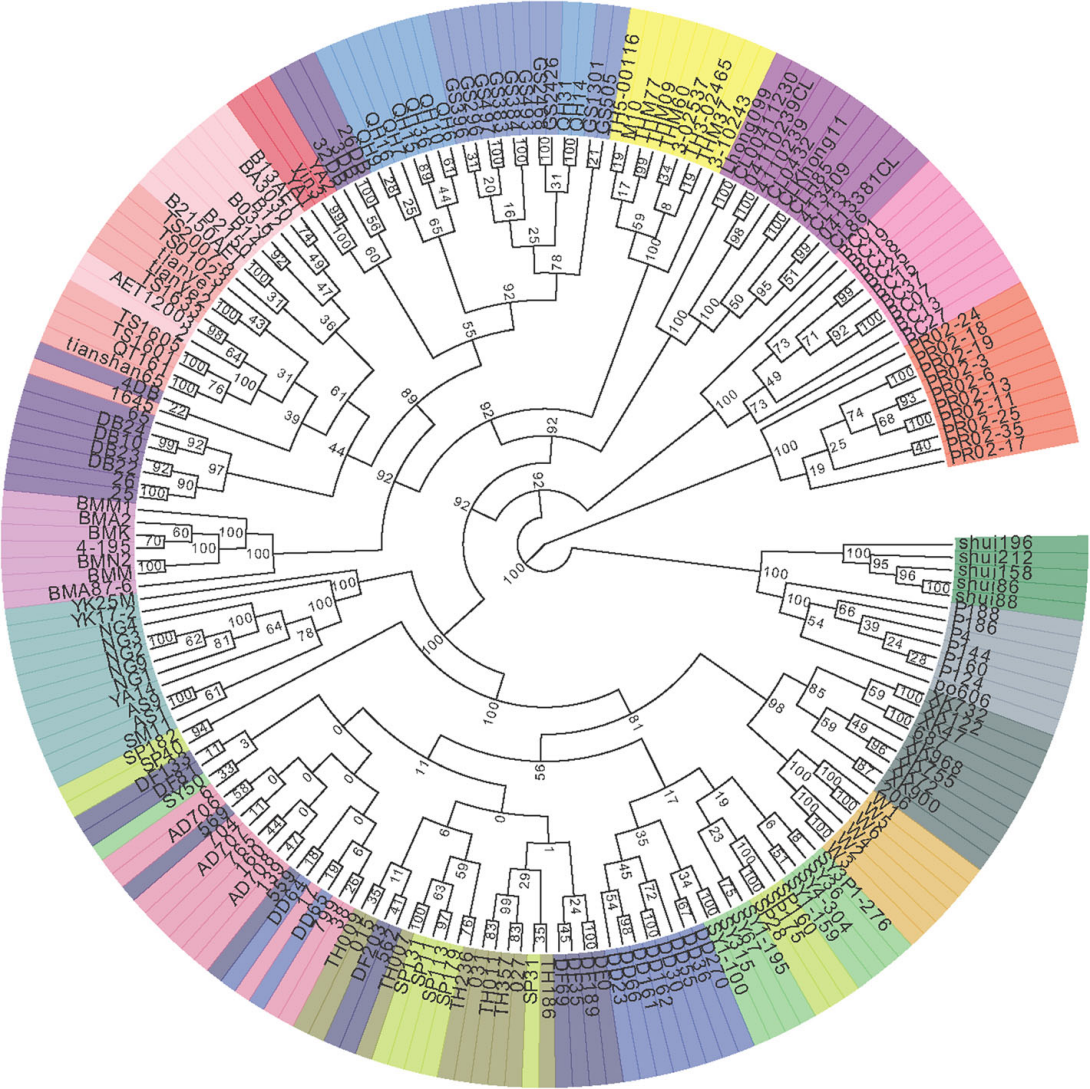
Phylogenetic ML tree of all *Cervus* species based on the individual SNP matrices. Phylogenetic analyses were conducted using maximum likelihood (ML) on individual SNP matrix. One colour for each group: *R. tarandus,* OrangeRed; *C. eldii,* LightSlateGray; *C. unicolor,* Green; *C. albirostris,* HotPink; *C. elaphus* (New Zealand), DarkMagenta; *C. e. yarkandensis,* Yellow; *C. c. songaricus,* LightCoral; *C. c. sibiricus,* LightPink; *C. c. xanthopygus,* Indigo; *C. c. macneilli,* DodgerBlue; *C. c. kansuensis,* RoyalBlue; *C. c. alashanicus,* Crimson; *C. c. canadensis*, Orchid; *C. nippon* (shuangyang), SpringGreen; *C. nippon* (tonghua), Olive; *C. nippon* (dongda), Blue; *C. nippon* (dongfeng), MidnightBlue; *C. nippon* (siping), GreenYellow; *C. nippon* (aodong), PaleVioletRed; *C. nippon* (ussuri), Goldenrod; *C. nippon* (xingkaihu), DarkSlateGray; *C. n. aplodontus,* CadetBlue. Phylogenetic ML tree of all *Cervus* species based on the individual SNP matrices was basically the same as inter-population combined analysis, but some of the samples fell within their respective groups as anticipated, such as *C. c. songaricus* and *C. c. sibiricus, C. c. macneilli* and *C. c. kansuensis* (these populations do have overlapping ranges), *C. c. xanthopygus* was split to 2 populations, one of which has close relationships with *C. c. sibiricus* and *C. c. songaricus*, and the other was grouped with *C. c. alashanicus*. Similarly, the *C. nippon* populations except *C. n. aplodontus* could not be well-separated. The phylogenetic ML tree of all *C. elaphus* and *C. canadensis* based on the individual SNP matrices, together with geographic distribution, have showed that, there were three major subclades in China, namely *C. e. yarkandensis* (distributed in Tarim Basin, with 92% bootstrap support), *C. c. macneilli/C. c. kansuensis/C. c. alashanicus* (distributed in middle west of China, with 89% bootstrap support), and *C. c. songaricus/C. c. sibiricus* (distributed in northwest and northeast of China, with 89% bootstrap support)

Phylogenetic ML tree of all *Cervus* species based on the individual SNP matrices was basically the same as inter-population combined analysis, but some of the samples fell within their respective groups as anticipated (Fig. 4), such as *C. c. songaricus* and *C. c. sibiricus*, *C. c. macneilli* and *C. c. kansuensis* (these populations do have overlapping ranges), *C. c. xanthopygus* was split to 2 populations, one of which has close relationships with *C. c. sibiricus* and *C. c. songaricus,* and the other was grouped with *C. c. alashanicus*. Similarly, the *C. nippon* populations except *C. n. aplodontus* could not be well-separated. These results were probably caused by no geographical isolation between these local populations.

The phylogenetic ML tree of all *C. elaphus* and *C. canadensis* samples based on the individual SNP matrices, together with geographic distribution, have showed that, there were three major subclades in China, namely *C. e. yarkandensis* (distributed in Tarim Basin, with 92% bootstrap support), *C. c. macneilli/C. c. kansuensis*/*C. c. alashanicus* (distributed in middle west of China, with 89% bootstrap support), and *C. c. songaricus*/*C. c. sibiricus* (distributed in northwest and northeast of China, with 89% bootstrap support), whereas there might be introgression in the current *C. c. xanthopygus* population (Figs. 1 and 4). Among them, *C. e. yarkandensis* was molecularly the most primitive subclade, with a differentiation dating back to 0.8–2.2 Myr ago.

### Interspecific gene exchange analysis

**Fig. 5:**
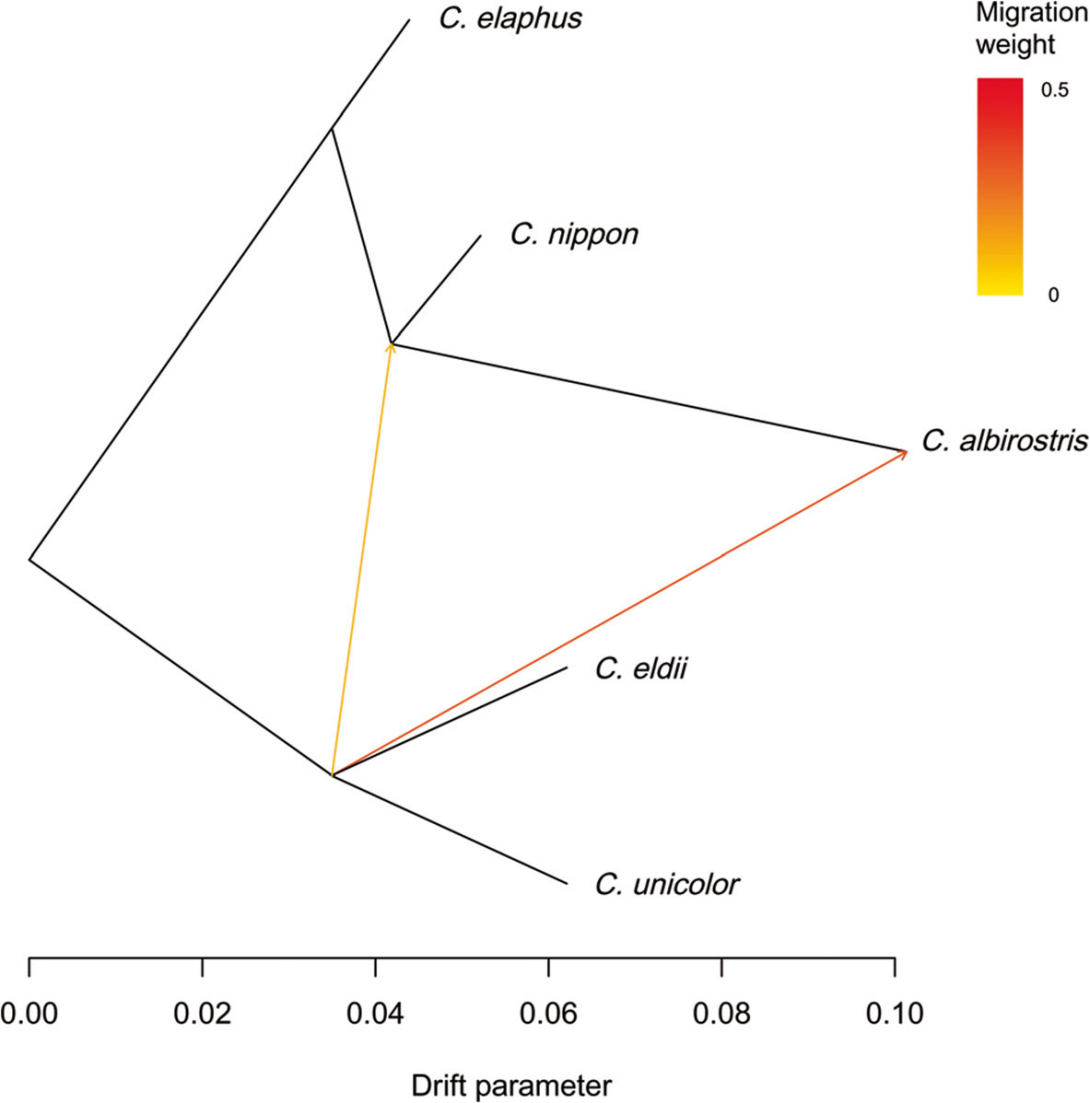
Migration events between species of the genus *Cervus* estimated by TreeMix. Population differentiation and mixing were estimated by TreeMix based on genome-wide allele frequency, 2–5 gradients were set respectively, combined with D statistical results, we found that there might be 2 migration events among 5 species in the genus *Cervus*. The direction of migration events are shown by arrows, and intensity is represented by color depth. Both migrations started from a common ancestor of *C. eldii* and *C. unicolor*, some of the ancestors migrated to *C. albirostris*, and another part migrated to the common ancestor of *C. nippon* and *C. albirostris*

Based on D statistical results, *p*-value was transformed to Z value, if the absolute value of Z is greater than 2 (namely *P*-value< 0.05), the higher the probability of interspecific genetic exchange events is. The result was showed in Table 3. Group I, II, III conformed to BABA model, indicating *C. albirostris* and *C. eldii*, *C. albirostris* and *C. unicolor* had more probability of interspecific genetic exchange; Group IV, V conformed to ABBA model, indicating *C. unicolor* and *C. albirostris*, *C. unicolor* and *C. nippon* had more probability of interspecific genetic exchange. Population differentiation and mixing could be explained by the estimation of migration events, which included both geographic migration and evolutionary process, and they might all be marked by the genetic evidences. In this study, migration events estimated by TreeMix was shown in Fig. 5, there might be 2 migration events among 5 species in the genus *Cervus*: both migrations started from a common ancestor of *C. eldii* and *C. unicolor*, some of the ancestors migrated to *C. albirostris*, and another part migrated to the common ancestor of *C. nippon* and *C. albirostris*.

**Table 3:** Interspecific genetic exchange estimated by D statistical analysis

	W	X	Y	Z	Z value
I	*C. albirostris*	*C. elaphus*	*C. eldii*	*R. tarandus*	2.638
II	*C. albirostris*	*C. nippon*	*C. eldii*	*R. tarandus*	3.754
III	*C. albirostris*	*C. nippon*	*C. unicolor*	*R. tarandus*	2.421
IV	*C. eldii*	*C. unicolor*	*C. albirostris*	*R. tarandus*	−2.143
V	*C. eldii*	*C. unicolor*	*C. nippon*	*R. tarandus*	−2.053

## Discussion

### Phylogeny and genetic diversity analysis using whole genome SNPs in the genus *Cervus*

In many studies, molecular markers were used to verify the accuracy of genetic resources [32–35], whereas species in the genus *Cervus* had close relationships, all the species could hybridize with each other. Little genetic markers could be used to disentangle phylogenetic relationships among the closely related species. A series of methods were used for these analysis, such as using more data to reconstruct phylogeny [36], or developing a gene-by-gene method [37], however, the amount of markers is still limited in these methods, and most markers were not suitable for inferring the species phylogenies [38]. Recently, the improvements of next generation sequencing based genotyping methods have made the classification and characterization of genetic resources more feasible. Large numbers of SNP markers could be generated and used to infer phylogenetic relationships among very closely related species. Based on these strategies, reduced-representation genome sequencing (RRGS) [39] was developed, such as reduced representation shotgun sequencing (RRS) [40], restriction site associated DNA sequencing (RAD) [41–44], genotyping by sequencing (GBS) [45] have been successfully used to infer phylogenetic relationships [46]. Among these methods, GBS is relatively simple and quick for phylogenetic studies [39]. The GBS protocol was first developed using one restriction enzyme [47] and then modified by using two restriction enzymes [48]. The two enzyme GBS approach could generate uniform complexity reduction and avoid sequencing of repetitive regions. GBS has been successfully applied in crops to resolve phylogenetic relationship and genetic diversity [49–51]. Therefore, reduced-representation genome sequencing has great potential for phylogenetic inference based on large data sets.

We report here the first genome-wide study on 21 subspecies/populations in the genus *Cervus*. The sequencing depth is enough for SNP screening and analysis [52]. All the subspecies/populations showed a certain degree of genetic diversity,whereas in *C. nippon*, *C. elaphus* and *C. canadensis*, the genetic varation was relatively low, this might be caused by the small population size and inbreeding effect. The result was accordant with other genetic diversity study of *C. nippon* and *C. elaphus* [53]. More importantly, the mapping of 5 species in the genus *Cervus* to the sika deer genome resulted in a high mapping rate, providing a useful data set for the study of genetic diversity and phylogeny of *Cervus* deer. In comparison to previous studies, which used mitochondrial DNA [4, 13, 14, 54] or microsatellite DNA [23, 24], the genome-wide data set is remarkable for its amount and quality of data [2], and the genomic results were close to the theoretical limit of uncertainty for the ages of mammalian ordinal and supraordinal clades [55].

### Phylogeny of closely related *C. elaphus, C. canadensis* and *C. nippon* complex

It is usually considered that *C. elaphus* and *C. canadensis* are not conspecific [7], they constitute three well-supported groups that are often ranked at species level: European and West-Asian red deer form a group, Central Asian red deer form a second group, and East Asian and North American wapiti form a third group [56, 57]. Our study do follow these opinions, in the *C. elaphus* clade, the divergence between *C. elaphus* (New Zealand) and the other occurred firstly, form one group; then the separation of *C. e. yarkandensis*, form a second group; and *C. c. canadensis* and other *C. canadensis* populations form a third group.

Interestingly, the phylogenetic position of the *C. e. yarkandensis* and *C. elaphus* (New Zealand) were surprising in this study, because they were sisters to the *C. canadensis* while usually *C. canadensis* turn out to be more closely related to *C. nippon* than to *C. elaphus* [56, 58]. *C. elaphus* (New Zealand) in this study are in all likelihood descendants of European red deer, while the New Zealand sample might also be derived from *C. c. canadensis*, but in this study, *C. elaphus* (New Zealand) were not clustered with *C. c. canadensis*, so our New Zealand samples could be the descendants of *C. elaphus*. The estimated divergence of this artificial population from the indigenous Eurasian *Cervus* has a little value and does not show a real chronology of evolutionary processes. Apparently, the obtained time of divergence at Pliocene or Early Pleistocene time is an overestimation and may be related to the founder effect, artificial selection and other genetic phenomena usual in the case of an artificial introduced population. Since the present data are genomic and not just mtDNA or single nuclear loci, this is potentially very interesting as it would restore *C. elaphus* and *C. canadensis* monophyly to the exclusion of *C. nippon*. In the following studies, European red deer samples (from Europe proper, covering the different phylogeographic lineages there) would be included to see whether this pattern holds.

### Subclades of *C. elaphus* and *C. canadensis* in China

Most of studies agreed with that the clade of *C. elaphus* and *C. canadensis* was divided to western and eastern subclades, the western subclade comprised the *C. e. yarkandensis* and the European populations, the eastern subclade consisted of *C. c. songaricus*, *C. c. sibiricus* and populations from other Asian areas (Alashan, Gansu, Tibet, Mongolia, and northeastern China), and *C. c. canadensis* [15, 18]. It was revealed that a boundary separating the western subclade from the eastern subclade occurs between Tarim Basin and Tienshan Mountains in China, *C. e. yarkandensis* was more closer to European red deer, meanwhile, *C. c. canadensis* were genetically closer to Asian populations in north China and Mongolia, supporting that *C. elaphus* might immigrated from northeastern Eurasia to North America through the glacier-induced land-bridge (Beringia) which had formed between the two continents after Late Pleistocene [57], this was accordant with our phylogenetic tree result. Based on our analysis of good quality and non-redundant SNPs, we suspected that *C. e. yarkandensis* might be a subspecies of European red deer, *C. c. canadensis* might originated from *C. elaphus* or *C. e. yarkandensis*, so they probably migrated from these regions. Other *C. canadensis* in China were clearly separated from *C. e. yarkandensis*, it might be interpreted by the independent evolution of *C. e. yarkandensis* in a relatively closed natural environment after they separated from other *C. elaphus* populations [14, 59].

The genetic analysis based on mitochondrial DNA cytb showed that, *C. c. kansuensis* and *C. c. macneilli* had the same genotypes [60, 61], we also found that samples from these populations were clustered as one group, it was indicated that these samples should be classified as *C. c. kansuensis*, the populations in Qinghai should not be considered as a subspecies. Other studies based on mitochondrial DNA D-loop regions found that there were gene exchanges between *C. c. sibiricus*, *C. c. songaricus* and *C. c. kansuensis*, probably caused by hybridization between populations [62], in the group we are studying also provided further evidence that there had been putative intra-subspecific crosses of *C. c. songaricus* and *C. c. sibiricus*, *C. c. xanthopygus* and other *C. canadensis*, however, difficult specimens in the genus may often be characterized as “hybrids” without adequate evidence of their hybrid status [63].

### Interspecific gene exchange of the genus *Cervus*

The genus *Cervus* had wide range of distribution. Every evolutionary type, from primitive to modern, were preserved during the evolution. Among them, *C. albirostris* is unique to China, it has similar karyotype to *C. unicolor* and *C. eldii* [64], the characteristics of their skulls are almost the same. Besides, their antlers also show continuous changes. In this study, interspecific gene exchange of the genus *Cervus* showed that *C. albirostris* had gene exchange with *C. unicolor* and *C. eldii*. It is consistent with previous research findings. *C. elaphus* and *C. nippon* are closely related, under natural conditions, they can hybridize with each other. But in this study, we didn’t find the evidence of gene exchange between the two species, we speculated that the similarity of gene frequencies between them is not necessarily due to the fusion effect of gene exchange, but to the same selection pressure. Gene exchange between species of the genus *Cervus* is a very interesting event, and it is still worth exploring in many different ways.

### Implications for conservation

Along with increasingly extensive and intensive human activities, the influence on *Cervus* deer is increasing gradually. Many subspecies in the genus *Cervus* are extinct in the wild (e.g. *C. n. taiouanus*, *C. n. grassianus* and *C. n. mandarinus*) [65]. Several species are listed as “Endangered” and “Vulnerable” by the IUCN (e.g. *C. eldii*, *C. albirostris*, *C. unicolor*), however, *C. elaphus*, *C. canadensis* and *C. nippon* were classified as “Least Concern” species, most of their subspecies are on the brink of extinction. As to *C. canadensis* in China, no distinction between *C. c. songaricus* and *C. c. sibiricus*, *C. c. macneilli* and *C. c. kansuensis* was evident from phylogenetic ML tree based on the individual SNP matrices. *C. c. xanthopygus* was split to 2 populations, one of which has close relationships with *C. c. sibiricus* and *C. c. songaricus,* and the other was grouped with *C. c. alashanicus*. Similarly, the *C. nippon* populations except *C. n. aplodontus* could not be well-separated. The conservation status of these populations will need to be reassessed, urgent protection measures were required to prevent introgression or extinction of these populations.

## Conclusions

The high-performance and low-cost of reduced-representation genome sequencing provides numerous high quality SNPs in the genus *Cervus*, enabled us to undertake phylogenetic analyses to answer the initial questions: (1) *C. unicolor* and *C. eldii* might be the most primitive *Cervus* deer. *C. albirostris* was the most closest to them with divergence time of 3.3–5.8 Myr ago. The split between *C. elaphus* and *C. nippon* could be estimated at around 3.6 millions of years ago. *C. elaphus*, *C. canadensis* and *C. nippon* might have the same ancestor. There were three major *C. elaphus* and *C. canadensis* subclades in China, within them, *C. e. yarkandensis* was molecularly the most primitive subclade, with a differentiation dating back to 0.8–2.2 Myr ago. (2) All 21 subspecies/populations had different degrees of genetic diversity. *C. eldii*, *C. unicolor* and *C. albirostris* showed relatively high expected and observed heterozygosity, while observed heterozygosity in *C. nippon* was the lowest, indicating there was a certain degree of inbreeding rate in these subspecies/populations. (3) There was high probability of interspecific gene exchange between *C. albirostris* and *C. eldii*, *C. albirostris* and *C. unicolor*, *C. nippon* and *C. unicolor*, and there might be 2 migration events among 5 species in the genus *Cervus*.

Our results provided new insight to the genetic diversity and phylogeny of *Cervus* deer. A revision of the current results based on comparison of phenotypic and molecular data is desirable for future research. In view of the current status of these populations, their conservation category will need to be reassessed.

## Additional files

Additional file 1: **Table S1.** Information on enzyme digestion results. (XLSX 21 kb)

Additional file 2: **Table S2.** Statistics of output, error rate, Q20, Q30, GC content of all the sequencing data. (XLSX 24 kb)

Additional file 3: **Table S3.** Summary of mapping rate and expected site coverage. (XLSX 29 kb)

## Abbreviations

*C. c.*: *Cervus canadensis*
*C. e.*: *Cervus elaphus*
*C. n.*: *Cervus nippon*
*C.*: *Cervus*
ddRADseq: Double digestion RADseq
GBS: Genotyping by sequencing
QC: Quality control
*R.*: *Rangifer*
RADseq: Restriction site associated DNA sequencing
RRGS: Reduced-representation genome sequencing
RRS: Reduced representation shotgun
SNPs: Single nucleotide polymorphisms
